# Supplementary material for: Multi-stakeholder perspectives regarding preferred modalities for mental health intervention delivered in the orthopedic clinic: a qualitative analysis
Source: BMC Psychiatry. 2023 May 19;23:347. doi: 10.1186/s12888-023-04868-9 (PMC10196288; doi:10.1186/s12888-023-04868-9)
Supplement: Supplementary file 1 — Supplementary Material 1 Semi-structured interview guide topics and questions [file 12888_2023_4868_MOESM1_ESM.docx]

**Additional file 1:** Semi-structured interview guide topics and questions

**Orthopedic Patients:**

**Digital mental health intervention**

1. This is an app that is specifically designed for people with chronic pain to help them manage their mental health. It uses a chatbot and text messaging with human counselors, and it can provide mindfulness and deep breathing exercises, counseling, journaling, and help with sleep (e.g., sleep stories, sleep sounds). [Provide complimentary access code for the participant to download and onboard to the app. Ask the participant to talk through their thought process as they download and onboard.]
   1. Usability:
      1. As you download the app and get started, what questions do you have? What is unclear?
      2. What are your thoughts as you try to talk to the chatbot? Use a tool pack?
      3. Can you show me how you would schedule a session with a human coach?
      4. Would you mind rating the app on this scale? [Complete System Usability Scale (SUS).]
   2. What do you think of the app?
      1. E.g., Design, appeal.
   3. What would you think if this app was introduced to you as part of your orthopedic care?
   4. How would you prefer the app to be offered?
   5. How much do you think you would use the app? Why?

**Printed mental health intervention**

1. We’re developing a wellness resource guide. The purpose of the guide is to provide patients with information about affordable local and online resources to address mental distress. [Show prototype.]
   1. What do you think of the guide?
      1. E.g., Design, appeal, name, information to add or remove.
   2. Usability:
      1. Can you show me how you would find and use a resource on the guide that looks interesting to you?
      2. Would you mind rating the guide on this scale so we can continue to improve it? [Complete System Usability Scale (SUS).]
   3. What would you think if this guide was introduced to you as part of your orthopedic care?
   4. How would you prefer the guide to be offered?
   5. How much do you think you would use the guide? Why?

**In-person mental health intervention**

1. What would you think about being offered in-person mental health support as part of your orthopedic treatment?
   1. If you think this would be a good option for some patients, how should it work?
      1. Referral vs real-time support in clinic?
      2. Social worker, licensed counselor, psychologist, or psychiatrist?

**Research considerations**

1. We’re thinking about conducting a research study to understand the helpfulness of providing mental health related treatment as part of an orthopedic care plan. What factors would play a role in whether you would be interested in participating in a study like this?
   1. Would you be willing to receive one of a few different treatment options (free of charge), by random chance?
   2. If we address all the factors you mentioned, do you think you would sign up for a research study like this?

**Other**

1. What other types of mental health related treatments should we try to offer in the orthopedic clinic?

**Orthopedic Clinical Team Members:**

**Digital mental health intervention**

1. These are screenshots from a mental health app that is specifically designed for people with coexisting chronic pain. It uses a chatbot and human counselors to deliver cognitive behavioral therapy, mindfulness techniques, and sleep tools (e.g., sleep stories, sleep sounds, and sleep hygiene techniques). [Show screenshots.] What are your thoughts regarding introducing an app like this in your outpatient orthopedic clinical practice as a mental health resource?
   1. What proportion of your patients do you think would be interested in trying this? And what would increase patient engagement with it?
   2. How would you envision this app fitting into your workflow?
   3. How much assistance (e.g., downloading, onboarding) could your clinical team provide when needed?
   4. What would be needed to achieve acceptability and buy-in from clinicians and staff to incorporate this into their practice?

**Printed mental health intervention**

1. We’re developing a printed wellness resource guide which describes details of local and online mental health resources. [Show prototype.] What are your thoughts regarding introducing a guide like this in your outpatient orthopedic clinical practice as a mental health resource?
   1. What proportion of your patients do you think would be interested in trying this? And what would increase patient engagement with it?
   2. How would you envision this guide fitting into your workflow?
   3. What do you think about the format of the guide for your patients?
   4. What would be needed to achieve acceptability and buy-in from clinicians and staff to incorporate this into their practice?

**In-person mental health intervention**

1. What are your thoughts regarding offering in-person mental health support for your orthopedic patients?
   1. What proportion of your patients do you think would be interested in trying this? And what would increase patient engagement with it?
   2. How would you envision in-person support fitting into your workflow?
      1. Referral vs real-time support in clinic?
      2. Social worker, licensed counselor, psychologist, or psychiatrist?
   3. What would be needed to achieve acceptability and buy-in from clinicians and staff to incorporate this into their practice?

**Research considerations**

1. We’re assessing the feasibility of conducting a fully powered randomized controlled trial to determine the effectiveness of mental health interventions for orthopedic patients. What factors would play a role in whether you would facilitate participation of your patients in a study like this?
   1. Do you think your patients would agree to participate? Be randomized?
   2. What workflow considerations would increase the feasibility and success of conducting a study like this?

**Other**

1. What other types of mental health interventions should we consider offering in the orthopedic setting?
